# Supplementary material for: Nonlinear dose-response association of moderate-to-vigorous physical activity with hyperuricemia in US adults: NHANES 2007–2018
Source: PLoS One. 2024 May 23;19(5):e0302410. doi: 10.1371/journal.pone.0302410 (PMC11115305; doi:10.1371/journal.pone.0302410)
Supplement: S1 Table — Abbreviations: BMI, body mass index. a analyses were adjusted for age, sex, education, race/ethnicity, ratio of family income to poverty, smoking status, alcohol drinking and BMI except the stratification variable. MVPA, moderate-to-vigorous physical activity per week(minutes). *: p < 0.05. (DOCX) [file pone.0302410.s001.docx]

**S1 Table. Stratified and Interaction Analyses for the Association of MVPA Levels with HUA^a^**

|  | **Odds ratio (95% CI)** | | | |
| --- | --- | --- | --- | --- |
|  | **MVPA (MET-min/wk)**  **600-2999** | **MVPA (MET-min/wk)**  **＜600** | **MVPA (MET-min/wk)**  **≥3000** | ***P* for interaction** |
| N | 8740 | 11839 | 8161 |  |
| Sex |  |  |  |  |
| Men | 1 [Reference] | 1.11 (0.95-1.29) | 0.96 (0.83-1.12) | **0.01** |
| Women | 1 [Reference] | 1.16 (0.99-1.36) | 1.12 (0.92-1.35) |  |
| Age |  |  |  |  |
| 20-29 | 1 [Reference] | 1.15 (0.85-1.54) | 0.96 (0.75-1.24) | 0.14 |
| 30-39 | 1 [Reference] | 1.00 (0.78-1.28) | 0.85 (0.64-1.12) |  |
| 40-49 | 1 [Reference] | 1.19 (0.89-1.60) | 1.20 (0.90-1.60) |  |
| 50-59 | 1 [Reference] | 1.22 (0.92-1.60) | 0.95 (0.70-1.29) |  |
| ≥60 | 1 [Reference] | **1.19 (1.00**-**1.42)*** | 1.07 (0.86-1.33) |  |
| Race/Ethnicity |  |  |  |  |
| Mexican American | 1 [Reference] | 1.23 (0.95-1.59) | 1.17 (0.88-1.57) | **0.01** |
| Other Hispanic | 1 [Reference] | 1.08 (0.83-1.40) | 0.84 (0.58-1.20) |  |
| Non-Hispanic White | 1 [Reference] | **1.22 (1.06**-**1.41)*** | 1.09 (0.96-1.24) |  |
| Non-Hispanic Black | 1 [Reference] | 1.06 (0.89-1.26) | 0.92 (0.75-1.12) |  |
| Other Race - Including Multi-Racia种族 | 1 [Reference] | 0.94 (0.73-1.21) | 0.92 (0.66-1.28) |  |
| BMI ,kg/m^2^ |  |  |  |  |
| ＜18.5 | 1 [Reference] | 1.52 (0.31-7.49) | 1.90 (0.39-9.25) | 0.37 |
| 18.5-24.9 | 1 [Reference] | 1.23 (0.93-1.64) | 1.28 (0.96-1.71) |  |
| 25-29.9 | 1 [Reference] | 1.02 (0.84-1.22) | 0.93 (0.76-1.13) |  |
| ≥30 | 1 [Reference] | **1.25 (1.08**-**1.43)*** | 1.04 (0.87-1.24) |  |

Abbreviations: BMI, body mass index.

^a^ analyses were adjusted for age, sex, education, race/ethnicity, ratio of family income to poverty, smoking status, alcohol drinking and BMI except the stratification variable.

MVPA, moderate-to-vigorous physical activity per week(minutes)

*: *p* < 0.05;
